# Prediction of the Seizure Suppression Effect by Electrical Stimulation via a Computational Modeling Approach

**DOI:** 10.3389/fncom.2017.00039

**Published:** 2017-05-29

**Authors:** Sora Ahn, Sumin Jo, Sang Beom Jun, Hyang Woon Lee, Seungjun Lee

**Affiliations:** ^1^Department of Electronic and Electrical Engineering, Ewha Womans UniversitySeoul, South Korea; ^2^Department of Neurology, Ewha Womans University School of Medicine and Ewha Medical Research InstituteSeoul, South Korea

**Keywords:** seizure suppression, electrical stimulation, computational model, *in vitro* experiment, seizure propagation mechanism, electrical field effect

## Abstract

In this paper, we identified factors that can affect seizure suppression via electrical stimulation by an integrative study based on experimental and computational approach. Preferentially, we analyzed the characteristics of seizure-like events (SLEs) using our previous *in vitro* experimental data. The results were analyzed in two groups classified according to the size of the effective region, in which the SLE was able to be completely suppressed by local stimulation. However, no significant differences were found between these two groups in terms of signal features or propagation characteristics (i.e., propagation delays, frequency spectrum, and phase synchrony). Thus, we further investigated important factors using a computational model that was capable of evaluating specific influences on effective region size. In the proposed model, signal transmission between neurons was based on two different mechanisms: synaptic transmission and the electrical field effect. We were able to induce SLEs having similar characteristics with differentially weighted adjustments for the two transmission methods in various noise environments. Although the SLEs had similar characteristics, their suppression effects differed. First of all, the suppression effect occurred only locally where directly received the stimulation effect in the high noise environment, but it occurred in the entire network in the low noise environment. Interestingly, in the same noise environment, the suppression effect was different depending on SLE propagation mechanism; only a local suppression effect was observed when the influence of the electrical field transmission was very weak, whereas a global effect was observed with a stronger electrical field effect. These results indicate that neuronal activities synchronized by a strong electrical field effect respond more sensitively to partial changes in the entire network. In addition, the proposed model was able to predict that stimulation of a seizure focus region is more effective for suppression. In conclusion, we confirmed the possibility of a computational model as a simulation tool to analyze the efficacy of deep brain stimulation (DBS) and investigated the key factors that determine the size of an effective region in seizure suppression via electrical stimulation.

## Introduction

Recently, deep brain stimulation (DBS) for refractory epilepsy has been receiving attention as an innovative treatment method. It is a method used to control epileptic seizures by directly applying an electrical stimulation to an epileptogenic lesion. Numerous clinical studies have shown a marked reduction in seizure frequency by DBS (Osorio et al., 2005; Boon et al., 2007; Fisher et al., 2010; Boëx et al., 2011; Valentín et al., 2013; Heck et al., 2014). However, it is still difficult to optimize the stimulation conditions for each patient, thereby limiting efforts to improve the efficacy of this therapy. Accordingly, research on DBS for epilepsy is actively pursuing multiple avenues simultaneously.

An experimental approach has been developed to assess the seizure suppression effect by electrical stimulation and identify its mechanism. Many groups have reported experimental results of *in vivo* and *in vitro* environments. *In vivo* experiments are largely carried out in chronic epilepsy models and have demonstrated alterations in seizure frequency or suppressive effects for on-going seizures due to stimulation (Wyckhuys et al., 2010; Rajdev et al., 2011; Rashid et al., 2012; Chiang et al., 2013; Cymerblit-Sabba et al., 2013; Huang and van Luijtelaar, 2014). *In vitro* experiments have mainly been conducted on brain slices using a bath application of convulsant drugs (Bikson et al., 2001; Lian et al., 2003; Schiller and Bankirer, 2007; Su et al., 2008; Jiruska et al., 2010). This method enables researchers to measure changes in specific ion concentrations, the characteristics of single neurons, as well as the local field potential of neuronal networks in a precise location, thus making it possible to elucidate the mechanisms of stimulation. We have also reported *in vitro* experimental data that support a convincing mechanism for the seizure suppression phenomenon: a neuronal depolarization blockade due to the accumulation of extracellular potassium ions (Ahn et al., 2017).

Meanwhile, a computational approach through computer modeling and simulation has also been employed in recent years. Most computational studies have focused on understanding the etiology of epilepsy and replicating epileptiform activities (Fröhlich et al., 2010; Jiruska et al., 2013; Jirsa et al., 2014; Ahn et al., 2016; Wendling et al., 2016), while others have sought to describe seizure control effect and its mechanisms (Colic et al., 2011; Volman et al., 2011; Beverlin li and Netoff et al., 2013; Mina et al., 2013; Taylor et al., 2015; Liu et al., 2016). Especially, Taylor and Baier proposed a neural field model to mathematically understand seizure dynamics (Taylor and Baier, 2011), the proposed model predicted that spike-wave seizures can be successfully abated by single pulse stimulation when applying real-time estimation to find optimal stimulation parameters (Taylor et al., 2014, 2015). In addition, by using the modified Taylor's model considered disinhibitory function (Fan et al., 2015, 2016), Liu and her colleagues presented that the onset of seizures can be delayed by the enhanced GABA_A_ inhibition to excitatory population (Liu et al., 2016). Meanwhile, Beverlin li and Netoff proposed a neuronal network model composed of single neurons, which descried the desynchronization of seizure activity by high-frequency stimulation based on a synaptic depression mechanism (Beverlin li and Netoff et al., 2013). Lastly, Mina and her colleagues presented a macroscopic model that replicated the modulatory effect of epileptic activity according to stimulation frequency (Mina et al., 2013).

Various studies have indicated that electrical stimulation can control seizure activity. In particular, responsive stimulation, in which stimulation is applied to an epileptic region when seizure activity has been detected or predicted, is capable of suppressing on-going seizure activity, even though the suppression effect occurs stochastically or locally. Nevertheless, integrated studies, which merge the advantages of experimental and computational studies, have been insufficient to increase the efficacy of DBS and optimize the stimulation protocol for seizure suppression.

In this paper, we elucidated factors that can affect seizure suppression due to electrical stimulation. Specifically, we investigated how the size of the effective region changes in response to these factors under the same stimulation conditions. The effective region refers to an area where seizure activity is completely suppressed by local electrical stimulation. For the purposes of this study, we classified *in vitro* experimental data (Ahn et al., 2017) into two groups according to the size of the effective region. We then compared characteristics of seizure-like events (SLEs) including propagation delay, frequency spectrogram, and phase synchrony between the two groups. Subsequently, we conducted computational modeling based on biological mechanisms that can reproduce seizure propagation and suppression phenomena due to electrical stimulation. Through simulations in various environments, using the proposed model, we were able to predict important factors that affect seizure suppression by stimulation.

## Materials and methods

### Analysis of *in vitro* experimental data

#### Classification according to the size of the effective region

**Figure 1:**
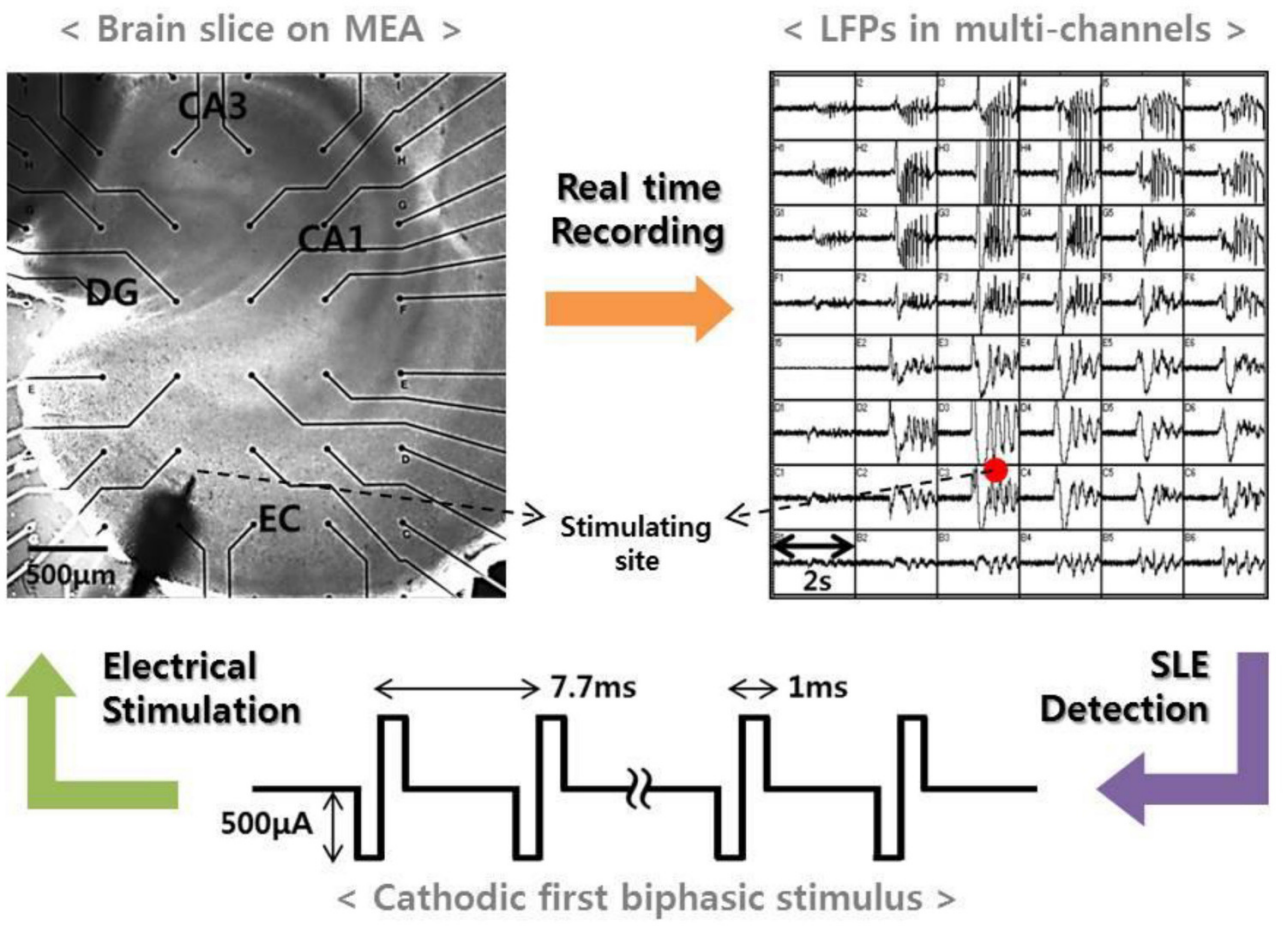
**Experimental method. (Left)** An EC-combined hippocampal slice on MEA. The slice consisted of sub-networks of EC, DG, CA3, and CA1. Black dots represent the recording electrodes, and the sharp point represents the stimulation electrode. **(Right)** Local field potentials recorded by electrodes corresponding to the positions shown in the figure to the left. The figure shows an abrupt SLE initiation and its propagation characteristics. When an SLE was clearly identified, a high-frequency stimulus with specific conditions (bottom figure) was applied within 5 s in order to suppress the synchronous activity.

Our previous study reported that electrical stimulation is able to suppress SLEs induced by a convulsant drug (Ahn et al., 2017). In the study, we continuously monitored local field potentials in entorhinal cortex (EC)-combined hippocampal slices of rats brains using a micro-electrode array (MEA). Then, we applied high-frequency stimulation to EC regions when a spontaneous SLE occurred and observed the changes in the network activities after the stimulation. Figure 1 presents a schematic of the experimental method. The upper two figures show an EC-combined hippocampal slice on an MEA and the field potentials recorded by each electrode at the onset of the SLE. The bottom figure shows the stimulus waveform we used with the following specifications: frequency, 130 Hz; pulse width, 1 ms; amplitude, 500 uA; duration, 3–5 s; and cathodic first biphasic rectangular pulses. A detailed experimental procedure is described in the previous paper (Ahn et al., 2017).

**Figure 2:**
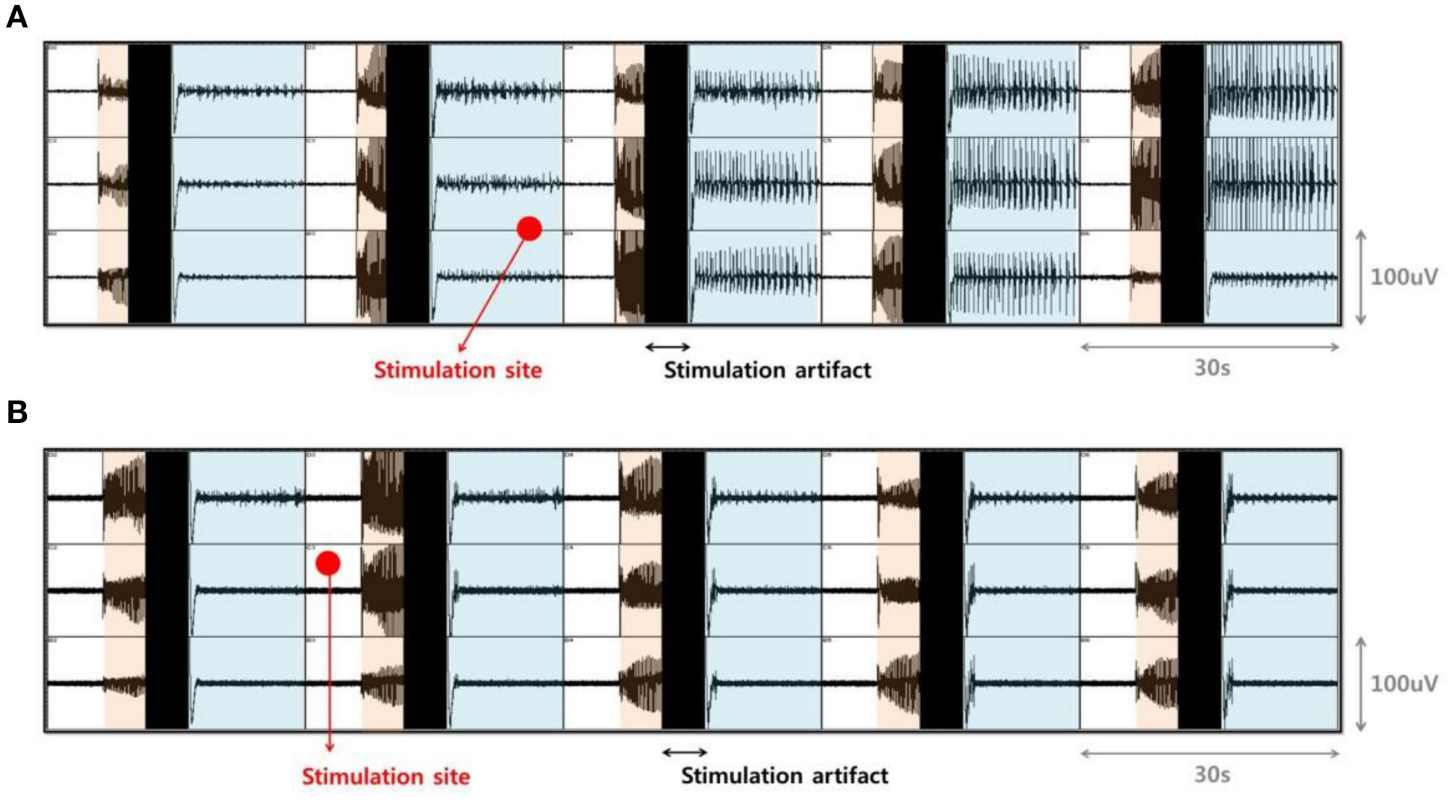
**Difference in the size of the effective region due to electrical stimulation. (A)** Examples from the “local EC suppression” and **(B)** “whole EC suppression” groups. Signals in each box were recorded by MEA simultaneously in the EC area. Orange-shaded regions show the generation of abrupt SLEs, black regions denote simulation artifacts, and the blue-shaded regions depict changes in local field potentials after stimulation. Red circles represent the positioning of the external stimulation electrode. Electrical stimulation was able to suppress on-going SLEs, even though the sizes of the immediately suppressed areas varied. After stimulation, only SLEs near the stimulating site were suppressed, while SLEs in other regions persisted **(A)**. SLEs in whole EC areas were completely suppressed simultaneously **(B)**.

In our previous study, electrical stimulation showed a mostly local suppressive effect; however, during bicuculline (BCC) bath application, the size of the effective region, where SLEs could be completely suppressed, varied (Ahn et al., 2017). Specifically, in ~50% of trials, only SLEs in the part of the EC region near the stimulating site were suppressed (Figure 2A). On the other hand, in ~25% of trials, all of the SLEs within the EC region, including those that were quite far from the stimulation site, were completely suppressed, despite using the same stimulation conditions (Figure 2B). Therefore, we aimed to explain which factors affected the size of the effective region. We first preferentially sorted 20 clear data samples recorded in different slices; 8 samples from the “whole EC suppression” group and 12 samples from the “local EC suppression” group.

#### Analysis of the SLE characteristics of the two groups

First, we assumed that the SLE characteristics between the two groups would be different. Thus, we analyzed them in terms of three different measures using MATLAB software (2015b, Mathworks). We used 3–5 s of SLE data prior to stimulation from each of the samples.

##### Propagation delay

SLEs were initiated by an abrupt change in the field potential and entered a stable state within 1–2 s, which was comprised of regular spikes with a frequency of 5–10 Hz. We chose three adjacent electrodes from the same layer of EC and calculated the propagation delays between them based on the peak time differences. We selected multiple peaks between 2 s and 3 s from the time of onset and used the average peak time differences to represent the propagation delay.

##### Frequency spectrum

We applied the spectrogram function in MATLAB in order to obtain short-time Fourier transform (STFT) results, which showed continuous results in the time and frequency domains. For the analysis, we used a 256 Kaiser window with an 85% overlap and a 2^13^ discrete Fourier transform (DFT) length for high resolution. Additionally, we calculated the mean power according to specific frequency bands of δ, θ, α, β, and γ, so as to confirm the dominant frequency band for the SLEs in each slice.

##### Phase synchrony

We calculated the phase locking value (PLV) to investigate the level of synchronization. The PLV is defined as a value between 0 and 1 that represents the phase synchrony between two signals, with 1 signifying complete synchronization (Lachaux et al., 1999). For the analysis, we chose the signals from four adjacent electrodes within the same layer of EC and narrowly filtered the signals at frequencies corresponding to each band (δ, θ, α, β, and γ). Next, the phases of the filtered signals were calculated by the Hilbert transform, and we determined the PLV by averaging the exponential values of the phase differences between 6(_4_*C*_2_) signal pairs according to Equation (1):

(1) $$PLV_{t}\;=\;\left|\frac{1}{N}\sum e^{i[\varphi_{j}\left(t\right)\;-\;\varphi_{k}(t)]}\right|$$

### Computational modeling

**Figure 3:**
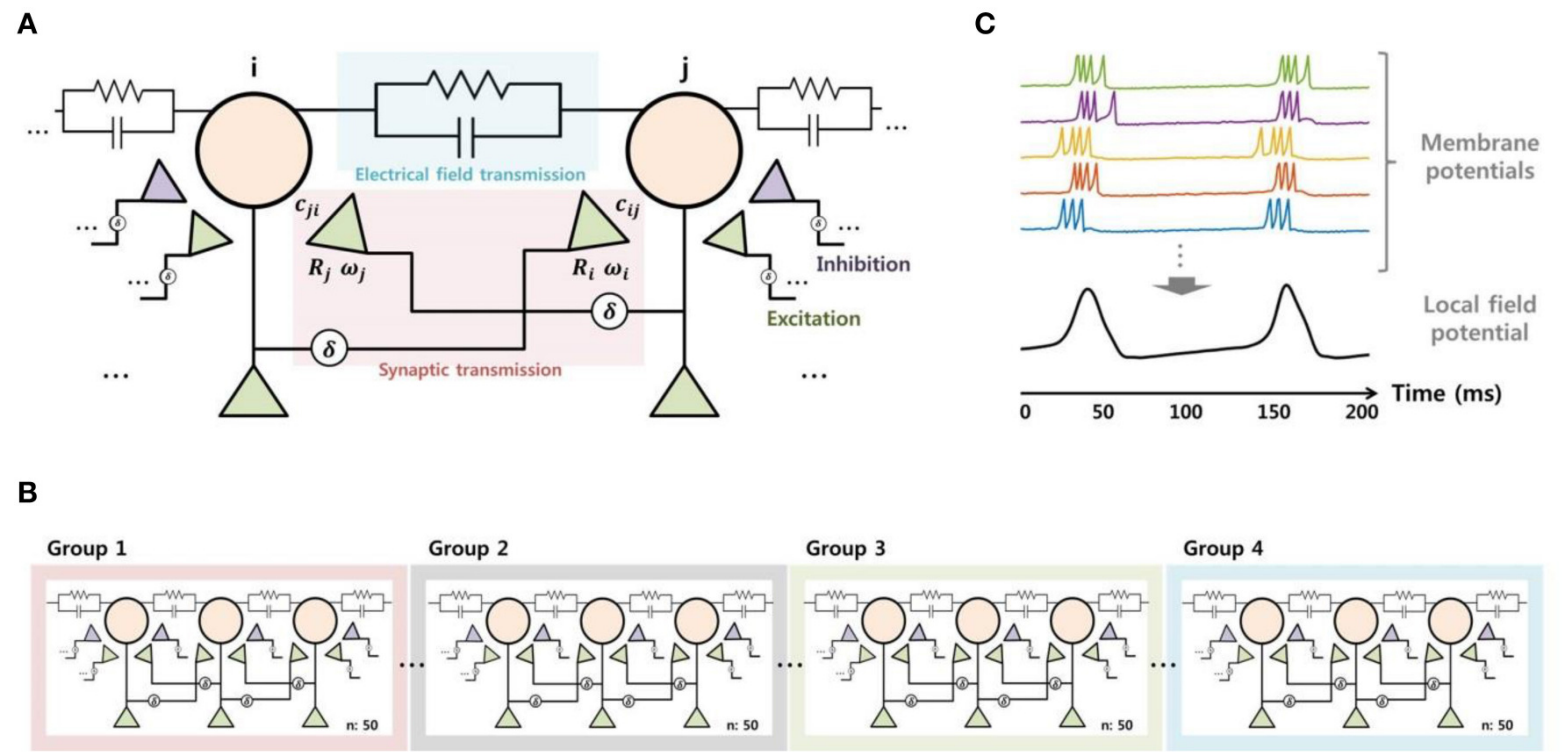
**Modeling of the signal transmission between neurons (A)**, network structure **(B)**, and the local field potential **(C)**. To model neuronal signaling, both the synaptic and electrical field transmissions were considered. Each principle neuron received synaptic current from five excitatory neurons over short and long distances and two inhibitory neurons. Also, the neurons received some current through electrical field transmission, which was proportional to membrane currents of neighboring neurons. The entire network was composed of 200 excitatory neurons and 40 inhibitory neurons, based on a small-world network structure. Meanwhile, the local field potential was obtained from the integration of neuronal activities. We calculated the average of the membrane potentials of 50 neurons after shifting, then applied low-pass filtering with a 50-Hz cutoff frequency to model the local field potential.

We constructed a neuronal network model using MATLAB in order to reproduce the SLE suppression effect due to electrical stimulation observed in *in vitro* experiments and to predict important mechanisms. We modeled a small-world network (Netoff et al., 2004), which consisted of 200 excitatory neurons and 40 inhibitory neurons. The characteristics of the neurons were replicated using Izhikevich's model (Izhikevich, 2003), which can reproduce various forms of neuronal activity with relatively simple computation. We selected parameters that enabled the description of spiking and bursting activity in excitatory neurons and fast spiking activity in inhibitory neurons, including a small perturbation for heterogeneity (Izhikevich, 2003; Izhikevich et al., 2004). To model signal transmission between neurons, we considered not only synaptic transmission (Izhikevich et al., 2004), but also electrical field transmission via the endogenous field (Fröhlich and McCormick, 2010; Qiu et al., 2015) (Figure 3A). The synaptic current consisted of conductance-based AMPA, NMDA, GABA_A_, and GABA_B_ currents and was controlled by short-term and long-term plasticity. The detailed equations are described in a previous paper (Ahn et al., 2016). Meanwhile, the electrical field induced by a change in the membrane voltage of some neurons can affect the activity in surrounding neurons through volume conduction. Also, the effect can be even stronger in the case of SLE because the field strength due to hyper-synchronization is stronger than in the normal state. A recent study reported that the electrical field transmission plays an important role in the propagation of epileptiform activity (Zhang et al., 2014). Thus, we modeled the electrical field effect by adding a small current to the neuronal input, which was determined instantaneously by the change in the membrane voltage of neighboring neurons. As a result, the input current of a single neuron is represented by Equation (2), where *I*_*syn*_ represents the synaptic current, which is the sum of the excitatory and inhibitory currents of pre-synaptic neurons. The *I*_*field*_ indicates the current due to the electrical field effect, which is proportional to the membrane current over the distance from other neurons (Qiu et al., 2015). The α symbol represents a parameter that controls for field transmission strength.

(2) $$\begin{array}{l} \text{I}=I_{syn}\;+\;I_{field}\\ I_{syn}=\sum I_{AMPA}+\sum I_{NMDA}+\sum I_{GABA_{A}}+\sum I_{GABA_{B}}\\ I_{field}=\alpha \sum \frac{I_{m}}{r} \end{array}$$

Figure 3B shows the network structure of the principle neurons. We induced SLEs by applying abrupt random inputs to part of neurons in group 1 while blocking the GABA_A_ current in order to replicate the experimental BCC effect. Neuronal activities initiated by the trigger input were synchronized spontaneously and propagated to other groups by signal transmission between neighboring neurons. Then, we modeled the SLE suppression effect by electrical stimulation based on a neuronal blockade mechanism via the accumulation of extracellular potassium ions. The detailed modeling method for this mechanism will be described in the Results section along with the simulation data.

Finally, we modeled the local field potential by integrating the membrane potential of neurons in order to directly compare the simulation results with the experimental data. We obtained an average of 50 excitatory neurons' membrane potentials after appropriate adjustment to approximately 0 mV. We were then able to acquire voltage signals in a manner similar to the recording data by calculating the average with 50-Hz low-pass filtering (Figure 3C). Consequently, the four local field potentials modeled from each group could be directly compared with the recorded data from four adjacent electrodes.

## Results

### Comparison of SLE characteristics between the two groups

We conducted data analysis to identify differences in the SLE characteristics between the two groups using three different features: propagation delays, frequency spectrum, and phase synchrony.

#### Propagation delay

**Figure 4:**
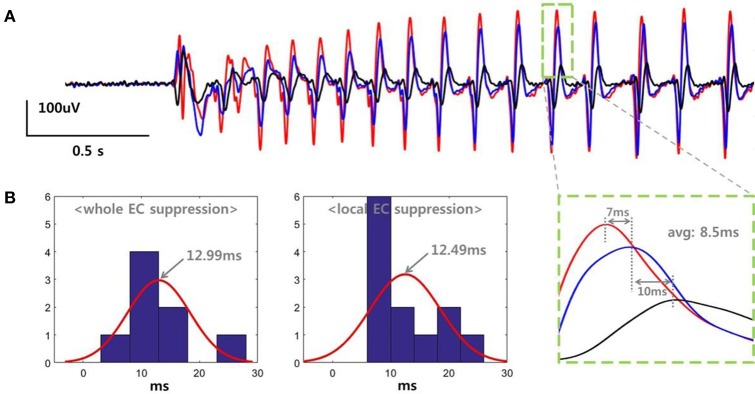
**SLE propagation delays in the “whole EC suppression” and “local EC suppression” groups. (A)** Signals recorded from three adjacent electrodes. The SLEs were initiated by an abrupt change in the field potential, followed by a stable state characterized by regular waveforms. The single positive peak that occurred during the stable state showed an average time delay of 8.5 ms. We calculated the SLE propagation delay by averaging the time differences of several peaks during the stable state. **(B)** Histogram representation of the propagation delays. ~12.99 ms and ~12.49 ms delays were observed on average in the “whole EC suppression” and “local EC suppression” groups, respectively. There were no significant differences between the two groups despite the observed variations.

We determined the propagation delay for the SLE based on the peak time differences between three adjacent electrodes. Figure 4A shows the data recorded in a slice belonging to the “whole EC suppression” group. Each color represents voltage signals recorded simultaneously in three different electrodes within the same layer of EC at a distance of 500 um. The SLE, initiated by an abrupt change in the field potential, stabilized gradually, and each signal generated regular waveforms with uniform magnitudes. As shown in the expanded plot, time delays of 7 and 10 ms were observed when measuring the distance between the positive peaks of the signal pairs, with an average of an 8.5 ms delay between peaks. We calculated the propagation delays for each slice as the average time differences for several peaks during the stable state. Figure 4B depicts histograms for the propagation delays and a normally-distributed curve for each group; 8 values were used in the “whole EC suppression” group, and 12 values were used in the “local EC suppression” group. In the “whole EC suppression” group, the mean was 12.9944 ms, and the standard deviation was 5.0238 ms. In the “local EC suppression” group, the mean was 12.4925 ms, and the standard deviation was 5.7574 ms. There was no significant difference between the two groups in terms of propagation delay even though the time delays of the “local EC suppression” group were relatively short.

#### Frequency spectrum

**Figure 5:**
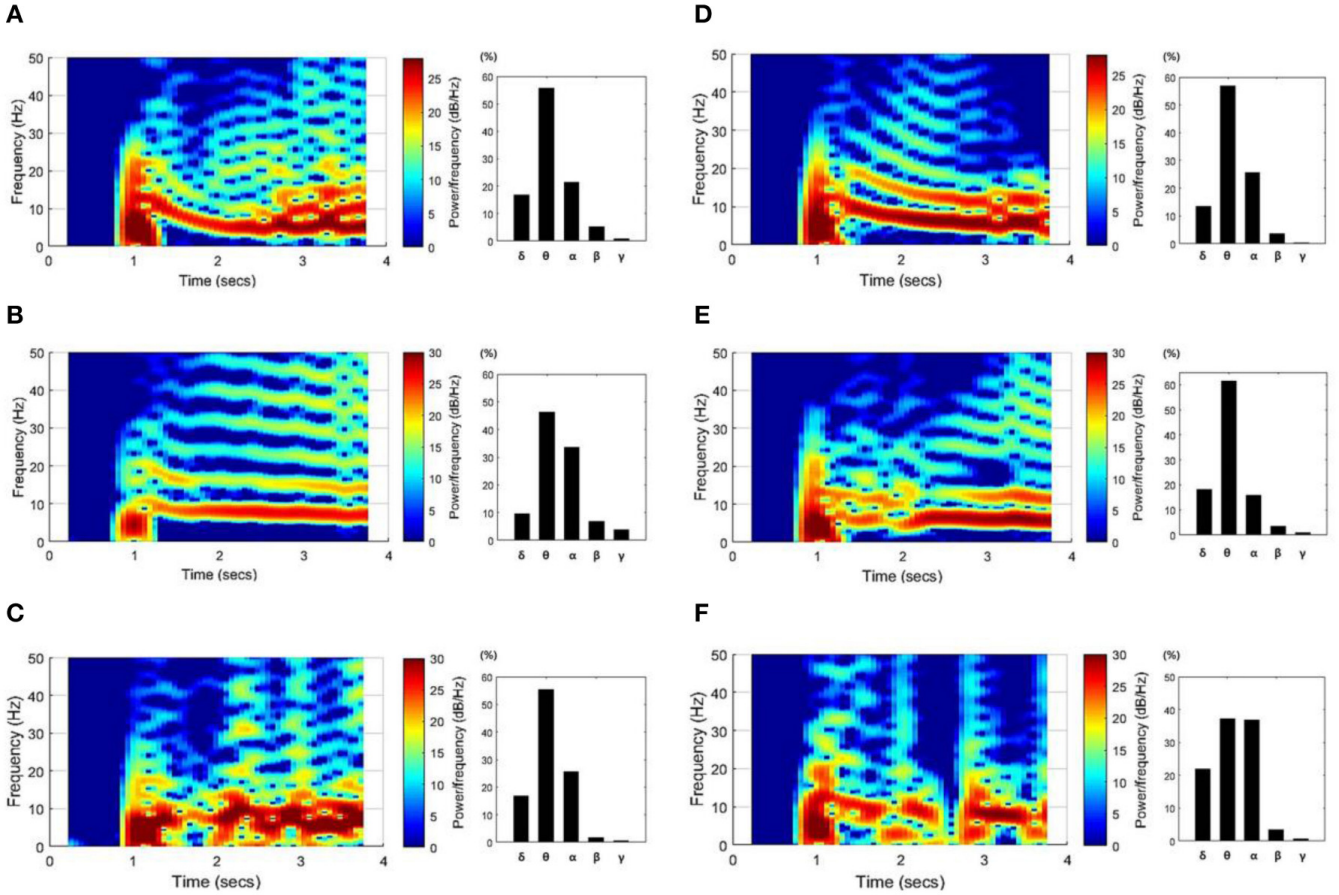
**SLE spectrograms and the mean power ratio per frequency band**. These figures represent the time-frequency characteristics of SLEs, which were derived using data ~3 s after onset. Results from the “whole EC suppression” group **(A–C)** and the “local EC suppression” group **(D–F)** are presented. In the spectrograms, the frequency components converged around the θ and α bands with time, even though a wider range of frequency components was observed in the SLE onset region. Meanwhile, the bar graphs show the percentage of mean power of each frequency band. The powers of the θ and α bands were relatively high, whereas the powers of the β and γ bands were extremely low. Considering the overall tendency, there was no marked difference between the two groups.

Figure 5 shows spectrograms of the SLEs from onset to ~3 s and the ratio of the mean power per frequency band. The left column results (Figures 5A–C) were obtained from the “whole EC suppression” group, and the right column results (Figures 5D–F) were acquired from the “local EC suppression” group. According to the spectrograms, most SLEs had a range of frequency components, from 0 to ~25 Hz, in the onset region. However, as time passed, the activities converged to mainly the θ or α band frequencies. Also, upon comparison of the average power during the first 3 s of each frequency band, the power of the θ and α bands constituted ~75%, whereas high-frequency components, corresponding to the β and γ bands, presented at a considerably lower percentage. Upon analysis of the frequency results in the two groups, we were unable to detect significant differences in the features of the spectrogram or in the mean power ratios.

#### Phase synchrony

**Figure 6:**
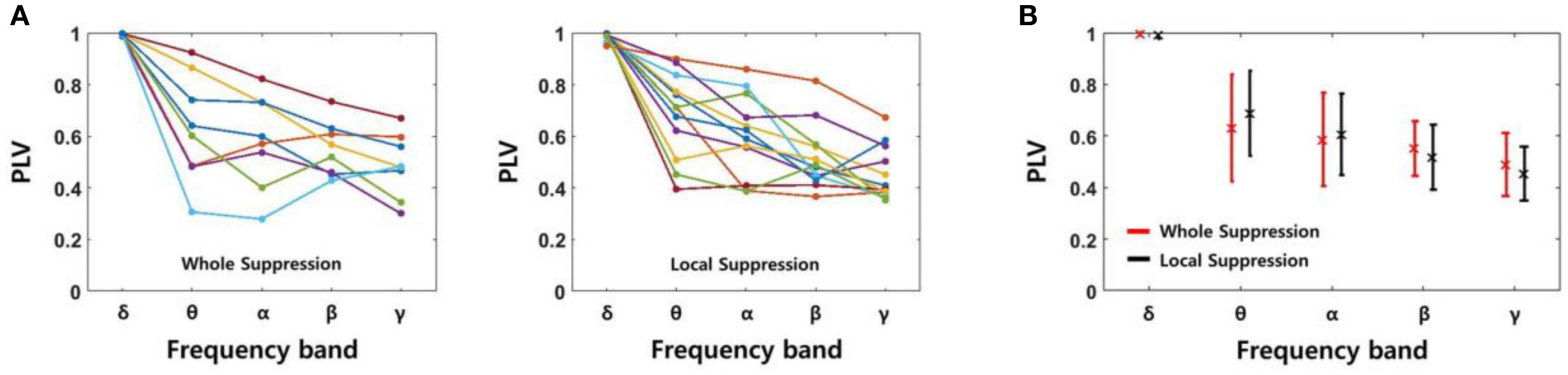
**PLVs of SLEs in each frequency band. (A)** The PLVs obtained from different slices in the “whole EC suppression” and “local EC suppression” groups, respectively. **(B)** The mean PLVs with standard deviations; red represents the whole suppression group, and black represents the local suppression group. The values of the δ to γ bands in sequence were 0.9952 (0.9895), 0.6308 (0.6864), 0.5844 (0.6044), 0.5499 (0.5172), and 0.4878 (0.4520), respectively, for the “whole EC suppression” group (“local EC suppression” group). A decreasing trend according to increase in frequency band was observed; i.e., higher-frequency components of SLE were less synchronized than low-frequency components. Comparison of the mean PLVs for the two groups did not yield any appreciable differences.

We determined the PLV using four signals recorded in the same layer of EC, i.e., six signal pairs were used for each slice. The PLV showed some fluctuation after the SLE was initiated. However, because the changes did not show a consistent trend, we calculated the average PLVs for 3 s starting at the time of onset. Figure 6A shows the PLVs in each frequency band, and different colors represent data obtained from different slices; 8 slices from the “whole EC suppression” group and 12 slices from the “local EC suppression” group. Also, Figure 6B presents mean PLVs for each frequency band in two groups. Although the PLVs from each data sample showed some variation, the PLVs acquired from higher frequency bands had lower values overall in both groups. The mean PLVs sequentially in the δ through γ bands were 0.9952, 0.6308, 0.5844, 0.5499, and 0.4878, respectively, in the “whole EC suppression” group. The mean PLVs in the same bands of the “local EC suppression” group were 0.9895, 0.6864, 0.6044, 0.5172, and 0.4520, respectively. These results suggest that higher-frequency SLE components, as measured in adjacent electrodes, were less synchronized than low-frequency components. Moreover, comparing the mean PLVs of all of the frequency bands did not reveal any distinct differences between the two groups.

### Prediction of the SLE suppression effect using a computational model

In the previous section, we investigated the differences in the SLE characteristics between the two groups. Nevertheless, the SLEs in the two groups did not show significant differences in any of the three aspects of propagation delay, frequency spectrum, or phase synchrony. Consequently, it was impossible to predict how large areas of SLE could be suppressed by local electrical stimulation by analyzing only SLE waveforms. Thus, we approached this question using a computational model to predict which factors affected the size of the effective region.

#### Modeling of the SLE suppression effect based on a biological mechanism

**Figure 7:**
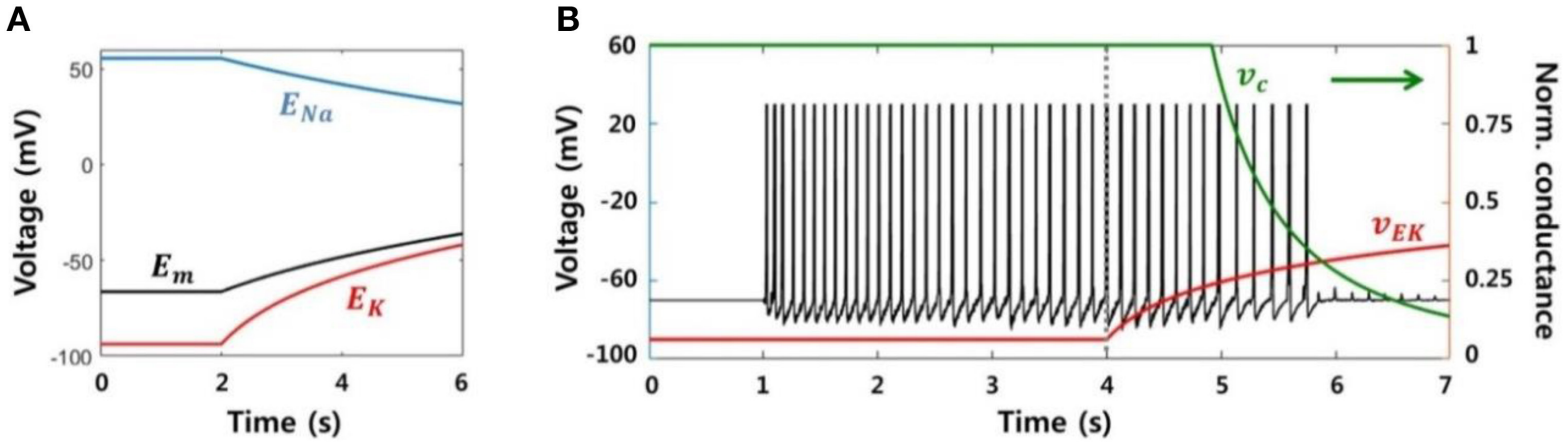
**Neuronal blockade due to an accumulation of extracellular potassium ions. (A)** Reversal potentials for each ion and the resting membrane potential. When a neuron generates a lasting action potential with a high frequency (after 2 s), the reversal potentials of the sodium and potassium ions decrease and increase, respectively, according to the influx and efflux of each ion. The resting membrane potential also increases due to the change in reversal potentials. **(B)** Simulation results of our model representing the activity of a single neuron considering the neuronal blockade mechanism. Abrupt trigger input and signaling between neighbors prompted the neuron to generate lasting burst activity with a frequency of ~8 Hz. However, the activity was gradually blocked by the application of the stimulation effect after 4 s, which resulted from a decrease in the neuronal input current dependent on *v*_*Ek*_.

We modeled a neuronal blockade due to an accumulation of extracellular potassium ions in order to replicate the suppression effect due to electrical stimulation. Assuming that a neuron fires continuously at a high frequency, the reversal potential of sodium ions (*E*_*Na*_) decreases and the reversal potential of potassium (*E*_*K*_) increases due to an influx and efflux of each ion, respectively. Figure 7A shows the change in the reversal potentials of each ion, as calculated by the Nernst equation, when the ion concentrations inside and outside of the neuron are linearly regulated. The resting membrane potential (*E*_*m*_) obtained via the Goldman-Hodgkin-Katz equation is also shown. Conductance of the sodium channels is reduced in response to an increase in the membrane potential or the potassium reversal potential, and this voltage dependence has been previously reported (Kim and Chung, 1999; Carlin et al., 2008). In other words, the sodium channel is inactivated, i.e., there is no inward flow of current, if the neuron is excessively depolarized.

In order to reflect this neuronal blockade mechanism in our model, first, we assumed that high-frequency stimulation makes neurons more excitable, leading to an accumulation of potassium ions in the extracellular region. We then added two other variables: *v*_*Ek*_, which represented the potassium reversal potential in the neuron, and *v*_*c*_, signifying the sodium conductance. The value of *v*_*Ek*_ increased logarithmically during the stimulation, and *v*_*c*_ decreased as a function of *v*_*Ek*_. Finally, we included *v*_*c*_ as a factor in the neuronal input current equation (2) to control the quantity of the input current. The modified equation was as follows:

(3) $$I=v_{c}(v_{Ek})\cdot (I_{syn}+I_{field})$$

Figure 7B presents the simulation result, the membrane potential of a single neuron. The neuron was activated by abrupt random input and generated sustained activities, with ~8 Hz of main frequency. Next, after 4 s, the stimulation effect was applied to the neuron by gradually increasing *v*_*Ek*_. Then, *v*_*c*_ was automatically reduced in accordance with *v*_*Ek*_. Although the neuron continuously received input at the same level, it could only accept a small amount of current due to *v*_*c*_. Consequently, spikes could not be generated after *v*_*Ek*_ was increased beyond a specific value.

The simulation results showed that SLEs can be suppressed by neuronal blockade from an accumulation of extracellular potassium ions. At the same time, they also implied that the suppressive effect would occur locally, i.e., only the neurons near the stimulating site that were strongly affected by the stimulation could be suppressed. However, in our previous experimental study, we observed that SLEs could be suppressed in a whole EC region, including areas far from the stimulation site, in a considerable number of slices under the same stimulation conditions. This indicates that a partial change in the network due to local stimulation could affect the entire network.

#### Prediction of the factors that affect the size of the effective region through network simulation

**Figure 8:**
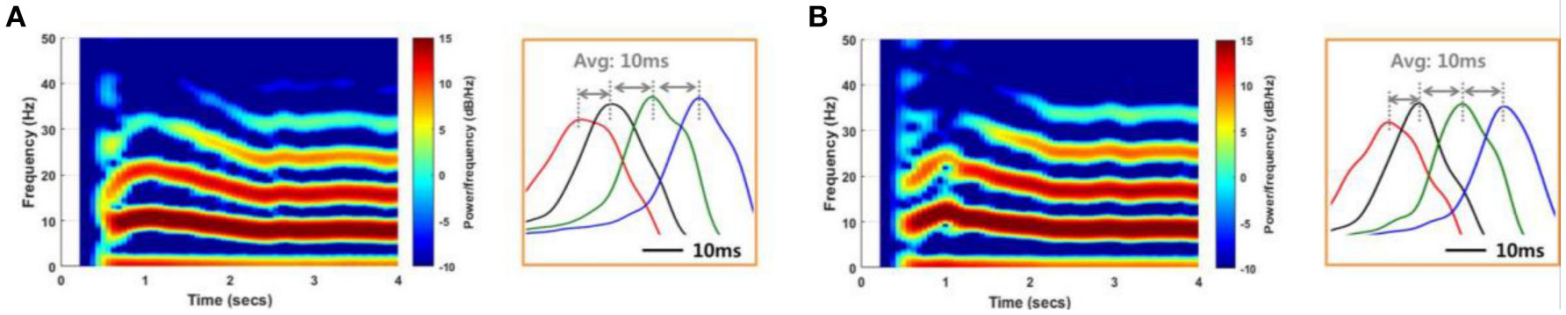
**Reproduction of SLEs by a computational model**. An SLE induced using an 85:15 ratio of synaptic current:field current **(A)** and another SLE induced using a 75:25 ratio **(B)** with a noise level of 1 μ*A*^2^. Spectrograms and the expanded plots of local field potentials for each SLE are shown. In both cases, the SLEs exhibited dominant frequencies of ~8 Hz and propagation delays of ~10 ms between groups, based on positive peaks differences. These results indicate that similar SLEs can be induced via different neuronal signal transmission mechanisms.

**Figure 9:**
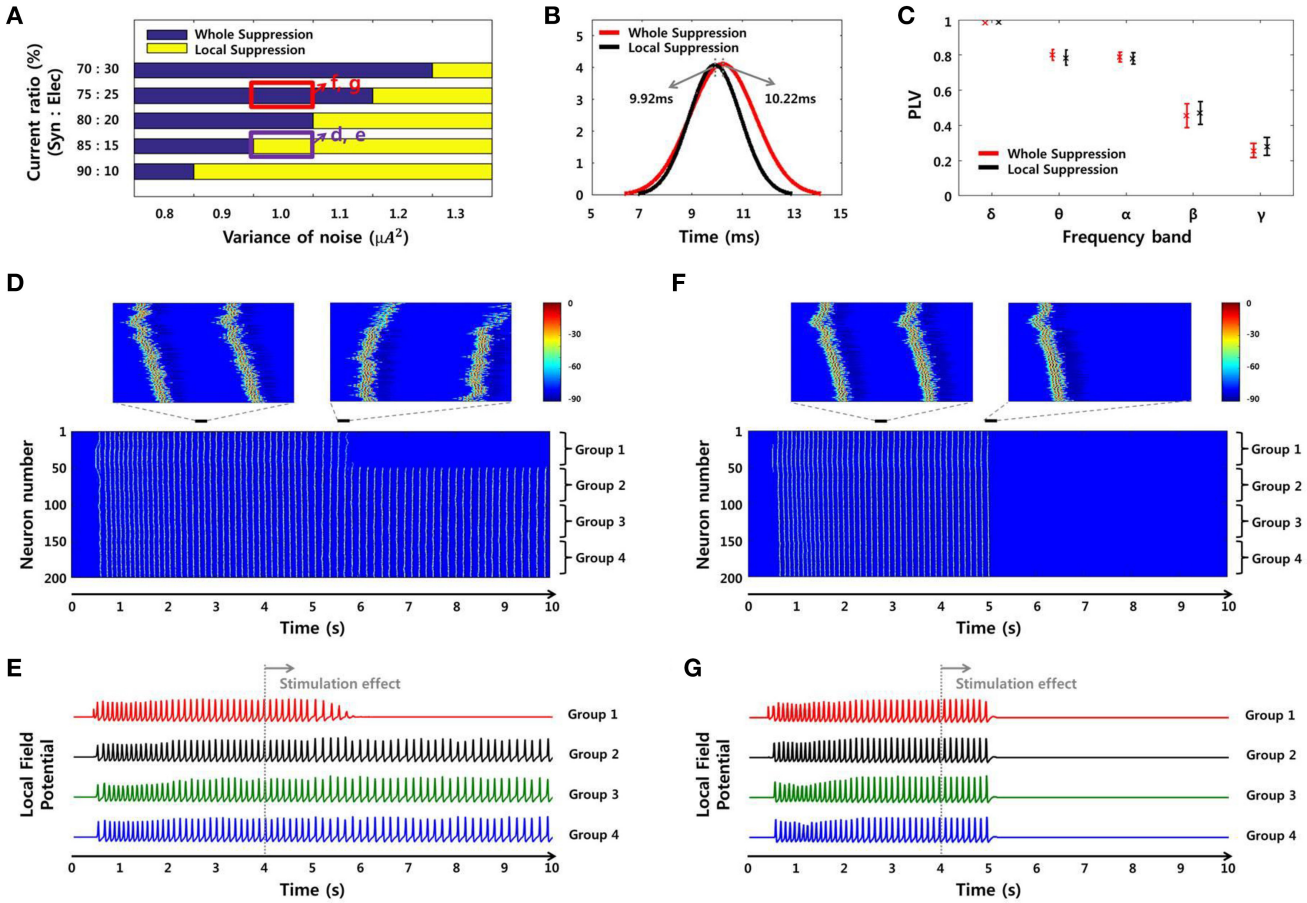
**SLE suppression effects through network simulation. (A)** The size of the suppressed region due to the local stimulation effect differed depending on noise level and SLE propagation mechanism. In the high-noise environment, only partial inhibition was observed, whereas in the low-noise environment, whole suppression was observed. In the same noise environment, the suppression effect was different according to the ratio of the two propagation mechanisms. As the ratio of electrical field current increased, whole suppression became prominent over local suppression. **(B–C)** Propagation delays and mean PLVs of simulated SLEs, classified by the size of the suppression region in **(A)**. The propagation delay was 10.22 ms (9.92 ms) on average, and the mean PLVs of the δ to γ bands in sequence were 0.9825 (0.9850), 0.7996 (0.7839), 0.7871 (0.7785), 0.4547 (0.4696), and 0.2562 (0.2792) in the whole suppression group (local suppression group). These results indicate that there was no appreciable difference in the signal characteristics between the two groups. **(D–E)** Neuronal activities and local field potentials in each group obtained from the 85:15 and **(F–G)** 75:25 ratios, with a noise level of 1 μ*A*^2^. When the stimulation effect was applied to neurons in group 1 at 4 s, neuronal activities in that region were suppressed after a delay in both cases. However, from the perspective of the whole network, the suppression effect occurred locally and only involved the group directly stimulated using the 85:15 ratio, while a global effect in the entire network was achieved using the 75:25 ratio.

We conducted a network simulation in order to compare the SLE suppression effects due to local stimulation under different conditions. First, we triggered an SLE by injecting ~1 s of abrupt random input into 25 excitatory neurons in group 1 (Figure 3B). The neuronal activities initiated by trigger input were spontaneously synchronized and propagated to neurons in other groups through signal transmission between neighboring neurons after a time delay (Figures 8, 9). During this signaling, both transmission by chemical synapses and the electrical field were involved simultaneously. We controlled the weight of the two transmission methods to replicate the SLE propagation. Specifically, we induced several SLEs having similar frequencies and propagation delays by maintaining a total input current similar to that of the neurons, but that differed in the ratio of synaptic and electrical field currents used. Ratios of synaptic current:electrical field current of 70:30–90:10 were used in order to simulate SLEs with similar characteristics. After the SLEs achieved ~3 s of stability, the stimulation effect was applied only to neurons in group 1, the initial group, to reflect a local stimulation effect. Then, we observed the changes in the activity of the entire network. We performed the same simulations in multiple noise environments.

Figure 8 shows spectrograms of two SLEs produced by the model from onset to 4 s. Figures 8A,B depict the results of the 85:15 and 75:25 synaptic current:electrical field current ratios, respectively, in which the noise variance was 1 μ*A*^2^. In both simulations, the main frequencies of the SLEs were ~8 Hz, and their spectrograms appeared quite similar to each other. The orange boxes display expanded plots of the local field potentials in each group, which is focused on a single peak in stable state. They show similar time delays between peaks; the synchronous activities initiated in group 1 (red) were propagated to other groups with an average delay of 10 ms. In addition, the mean PLVs sequentially in the δ through γ bands were 0.9863 (0.9815), 0.7912 (0.7809), 0.7901 (0.7636), 0.5403 (0.3697), and 0.2848 (0.2685) for the 85:15 ratio (75:25 ratio). Statistical data of multiple simulations are shown in Figures 9B,C. Consequently, SLEs with similar characteristics could be produced, despite being generated though different signal transmission mechanisms. Moreover, these results show that our model is able to replicate SLEs that include propagation characteristics similar to experimental data, allowing comparison between simulation results and measured data.

Although the SLEs had similar characteristics, their suppression effects differed in terms of the entire network. Figure 9A presents the suppression effect according to noise level and ratio of two SLE propagation mechanisms. At first, as the variance of noise was larger, the suppression effect was limited to the local network directly receiving stimulation. On the other hand, as the variance of noise was smaller, the suppression effect occurred throughout the entire network. In the same noise environment, the suppression effect was different depending on the ratio of propagation mechanisms. When the influence of electrical field transmission was very weak, only a local suppression effect was observed. However, when the effect of the electrical field transmission was a little stronger, partial inhibition due to local stimulation could modulate neuronal activities in the entire network; i.e., a whole suppression effect was observed. Figures 9D–G present the neuronal activities and local field potentials induced by each group using 85:15 and 75:25 ratios, respectively, in which the variance of noise was 1 μ*A*^2^. When the stimulation effect was applied to neurons in group 1 at 4 s, the neuronal activities in that region were suppressed after a delay in both simulations. The synchronous activities in the other groups were sustained regardless of the local suppression effect in Figures 9D,E, whereas they were completely suppressed simultaneously in Figures 9F,G. These results indicate that SLEs propagated by a strong electrical field effect can respond sensitively to partial changes in a network due to stimulation. In addition, these different simulation results can also explain our experimental data. They show that, even if basic environments including noise level are similar, the size of the effective region can be determined differently by the SLE propagation mechanism.

Meanwhile, Figures 9B,C show propagation delays and mean PLVs in each frequency band between the two groups, which were categorized according to suppression effect of simulated SLEs. They also confirmed that there was no significant difference in the signal characteristics between the two groups as in the experimental data analysis; 10.22 ms (9.92 ms) delays were observed on average, and 0.9825 (0.9850), 0.7996 (0.7839), 0.7871 (0.7785), 0.4547 (0.4696), and 0.2562 (0.2792) mean PLVs in the δ through γ bands, respectively, were derived from the whole suppression group (local suppression group).

**Figure 10:**
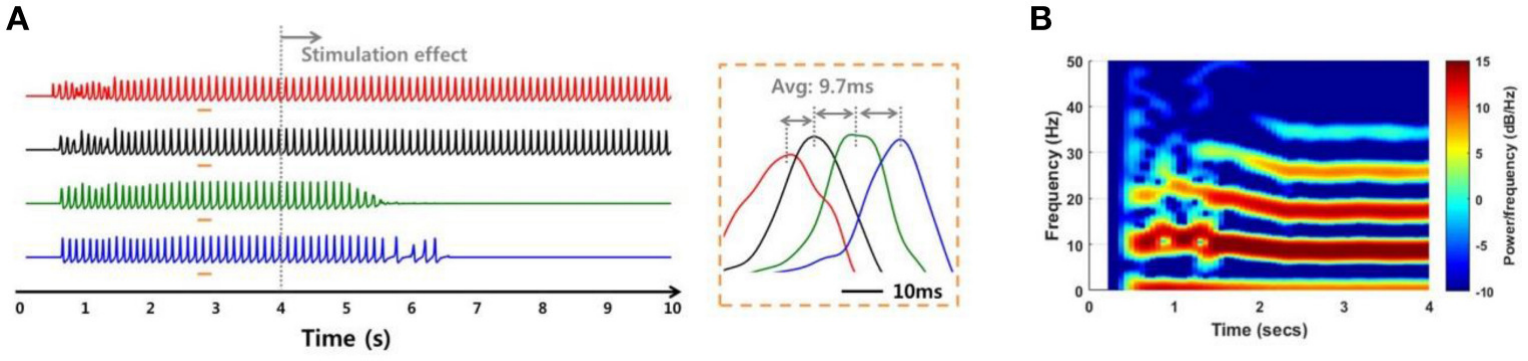
**Simulation results of an SLE suppression effect via applied stimulation to a non-focus region. (A,B)** Local field potentials and spectrograms for SLEs induced by a 75:25 ratio of synaptic current:field current. As in Figure 9, the SLE was initiated in group 1 (red) and propagated to other groups with a ~9.7 ms delay, with similar spectrogram results. When the stimulation effect was applied to neurons in group 3, which was not an SLE focus, only neuronal activities in groups 3 and 4 were suppressed, i.e., the activities in the previously initiated regions, groups 1 and 2, persisted irrespective of local suppression.

In addition to the internal environments such as noise and signal transmission mechanisms, the network simulation was able to investigate the SLE suppression effect according to external factors such as stimulation site. When the stimulation effect was applied to a non-initiating region, the suppression effect did not occur to the entire network, even in a low noise environment and even though the SLE was propagated by the stronger electrical field effect. Figure 10 shows a simulation result when the stimulation was applied to group 3 neurons (green) in which the variance of noise was 1 μ*A*^2^. The SLE was started in the group 1 neurons and propagated to the entire network consecutively, as shown in Figure 9. When group 3 neurons were blocked, the synchronous activities of the group 4 neurons (blue), which were propagated from group 3, were also suppressed over time, while the activities in other groups that were activated prior to group 3 were sustained. Simulation results applying the stimulation effect to group 2 or 4 were similar. This signifies that stimulating the focus region would be more effective for seizure suppression, which is also in accordance with previous experimental research (Chiang et al., 2013).

## Discussion

In this paper, we identified factors that can affect the size of the effective region in seizure suppression by electrical stimulation. After we classified *in vitro* experimental results into two groups, “whole EC suppression” and “local EC suppression,” we compared the SLE characteristics recorded in these groups. However, there was no significant difference between the groups in terms of propagation delay, frequency spectrum, or phase synchrony. In other words, the propagation characteristics and specific features of the SLEs themselves showed no obvious distinctions. This indicates that it would be difficult to predict the extent of seizure suppression expected with local stimulation applied to a particular region from an analysis of recorded signal waveforms from patients.

Using a computational approach, however, we were able to predict important factors. First, we built a neuronal network model with a small-world network structure. In the model, the SLEs were initiated in some part of the neurons by abrupt random input and BCC effect and were then propagated to other areas spontaneously by signal transmission to neighboring neurons. In order to model signaling between neurons, we considered both chemical synaptic connections and the electrical field effect. We were able to induce similar SLEs while differentially adjusting the contributions of these two methods, on multiple noise environments. The SLEs showed similar propagation delays and time-frequency characteristics. Also, the characteristics of the SLEs generated by the model were similar to experimental data. The simulation results indicate that the seizure activities initiated in a particular region can be propagated to other areas with similar patterns, despite different propagation mechanisms. This also confirms that the propagation speeds conducted through local synaptic connections and via electrical field effects are not significantly different, as noted in previous studies (Bao and Wu, 2003; Zhang et al., 2014; Fehérvári et al., 2015).

Although the SLEs had similar features, the suppression effects due to stimulation were distinct. At first, in the high-noise environment, the suppression effect was limited to the local network that directly received the stimulation effect. On the other hand, in the low-noise environment, the suppression effect occurred throughout the entire network including a region far from the stimulation site. These results can be interpreted to indicate that large noise can help sustain the synchronous activity by compensating for the reduction of input currents in neurons near the inhibited region. Meanwhile, interestingly, under the same noise environment, the size of the effective region was different according to the ratio of the two SLE propagation mechanisms. When the influence of electrical field transmission was very weak, only local suppression effects occurred. However, when the effect of the electrical field transmission was stronger, the suppression effect occurred globally to the entire network, i.e., partial suppression induced by local stimulation affected neuronal activities in the entire network. We speculate that these results are caused by differences in the characteristics of the two propagation methods. The effects of the electrical field are more instantaneous than those of synaptic transmission, which involve the chemical operation of a neurotransmitter. Thereby, if the neurons are synchronized by a stronger field effect, inhibition of part of a neuron can control the neuronal activities even in distant regions because the input current of neurons adjacent to the inhibited region is immediately reduced. Through these simulation results, we predict that the propagation mechanism of SLE can determine the size of an effective region by local stimulation. However, it is still unclear which conditions affect the propagation mechanism. This uncertainty might be due to multiple intrinsic properties of the network, such as connectivity, synaptic plasticity, and tissue conductivity. Future work to clarify these causalities should be conducted. Moreover, studies aimed at analyzing seizure propagation mechanisms in different patients are also important.

Additionally, another simulation using this model could also show the importance of the stimulating site. The results showed that stimulation of the focus region increases the size of the effective region due to local stimulation. Furthermore, this underscores the importance of localization studies to determine the precise seizure focus in each patient.

In this paper, we showed the possibility of a computational model as a simulation tool to analyze the efficacy of DBS. In particular, we showed the strength of a computational model by predicting important factors through simulation, which were not revealed by experimental results. In this model, we used a neuronal blockade mechanism in order to describe the SLE suppression effect by electrical stimulation. Especially, we modeled the blockade via the accumulation of extracellular potassium ions, which is considered the one of the convincing mechanisms to explain the suppression effect (Lian et al., 2003; Fröhlich et al., 2008; Ahn et al., 2017). Apart from this mechanism, several important mechanisms have been reported through clinical and experimental researches, they include synaptic inhibition, synaptic depression, and disturbance of pathological network activity (Dostrovsky et al., 2000; Montgomery et al., 2000; McIntyre et al., 2004; Schiller and Bankirer, 2007). In addition, a computational study has identified that different mechanisms could work depending on stimulus frequency (Mina et al., 2013). In the reported model, seizure suppression effect occurred by feedforward inhibition and short-term depression mechanisms in low-frequency stimulation, whereas it occurred by direct activation of interneurons that control excitatory neurons in high-frequency stimulation (Mina et al., 2013). Consequently, the working mechanism may differ according to neuronal network structure, signal pathways of target, causes of seizure activity, stimulus frequency used, etc. Thus, systematic studies are required to identify the appropriate mechanisms for each patient's condition and to derive optimum stimulation parameters through simulation.

Meanwhile, in this model, we focused on the dynamics of a small network, specifically, the EC region. However, our results can be applied to larger networks, such as the hippocampal network, which is composed of some sub-networks, or even whole-brain networks, considering the connections between the hippocampus and other parts of the brain. In medial temporal lobe epilepsy, it is well known that most seizure activity is generated in a specific part of the EC region and propagated to the entire EC area, other sub-networks of the hippocampus, and the other brain areas. Generalizing our findings to a larger network with moderate noise, only seizure activity propagated from the focus region via a strong electrical field effect would be completely suppressed by local stimulation of the focus region. However, the effect of synaptic transmission would be dominant in the propagation between the sub-networks or other brain areas because the effect of electrical field effect is rapidly reduced with distance, and relatively distant networks are connected by axon bundles. This indicates that global seizure suppression is difficult to achieve by local stimulation after considerable propagation, even if the stimulation is applied to the focus region. Thus, a system design that could rapidly detect seizure onset, identify the focus region, and automatically apply stimulation to the region would be very significant. In addition, simultaneous stimulation of multiple core sites obtained by data analysis may prove to be a more effective therapy.

## Ethics statement

This study was carried out in accordance with the ethical standards of Ewha Womans University. The protocol was conducted in accordance with the animal research guidelines of Use Committee of the Institute of Laboratory Animal Resources at Ewha Womans University (IACUC No. 15-045).

## Author contributions

SA, SBJ, HWL, and SL designed research; SA, SJ, SBJ, and HWL analyzed experimental data; SA performed modeling and simulation; SA and SL analyzed results and wrote the paper.

### Conflict of interest statement

The authors declare that the research was conducted in the absence of any commercial or financial relationships that could be construed as a potential conflict of interest.
